# RNA methyltransferase BCDIN3D is crucial for female fertility and miRNA and mRNA profiles in *Drosophila* ovaries

**DOI:** 10.1371/journal.pone.0217603

**Published:** 2019-05-30

**Authors:** Li Zhu, Susan E. Liao, Yiwei Ai, Ryuya Fukunaga

**Affiliations:** Department of Biological Chemistry, Johns Hopkins University School of Medicine, Baltimore, MD, United States of America; Universität Regensburg, GERMANY

## Abstract

RNA methyltransferases post-transcriptionally add methyl groups to RNAs, which can regulate their fates and functions. Human BCDIN3D (Bicoid interacting 3 domain containing RNA methyltransferase) has been reported to specifically methylate the 5′-monophosphates of pre-miR-145 and cytoplasmic tRNA^His^. Methylation of the 5′-monophosphate of pre-miR-145 blocks its cleavage by the miRNA generating enzyme Dicer, preventing generation of miR-145. Elevated expression of BCDIN3D has been associated with poor prognosis in breast cancer. However, the biological functions of BCDIN3D and its orthologs remain unknown. Here we studied the biological and molecular functions of CG1239, a *Drosophila* ortholog of BCDIN3D. We found that ovary-specific knockdown of *Drosophila* BCDIN3D causes female sterility. High-throughput sequencing revealed that miRNA and mRNA profiles are dysregulated in BCDIN3D knockdown ovaries. Pathway analysis showed that many of the dysregulated genes are involved in metabolic processes, ribonucleoprotein complex regulation, and translational control. Our results reveal BCDIN3D’s biological role in female fertility and its molecular role in defining miRNA and mRNA profiles in ovaries.

## Introduction

The epitranscriptome describes over 200 types of post-transcriptional RNA modifications, which can regulate the fates and functions of RNAs [1–3]. Post-transcriptional modification can occur on bases, riboses, and termini of RNAs. Dysregulation of RNA modification is associated with neurological disorders, cancers, and other diseases in human [4].

Among the many types of RNA modifications, methylation modification is the most prevalent. A wide variety of RNAs such as mRNAs, tRNAs, rRNAs, and small silencing RNAs, receive methylation modification on their bases, riboses, and termini, typically catalyzed by RNA methyltransferase enzymes that use S-Adenosyl methionine (SAM) as a methyl donor. For example, methylation of bases and caps of mRNAs can regulate their translation efficiency and stability [3, 5]. Methylation of bases and riboses in tRNAs can regulate their stability and codon-anticodon interaction [6]. Methylation of bases and riboses in rRNAs is important for efficient and accurate translation by ribosome [7]. Methylation of the 3′-terminal ribose of small silencing RNAs can stabilize them [8]. However, we are still far from the complete understanding of RNA methylation. Which RNAs on which positions are methylated by which RNA methyltransferase enzymes? What are the molecular and biological roles of each of these RNA methylation?

**Fig 1:**
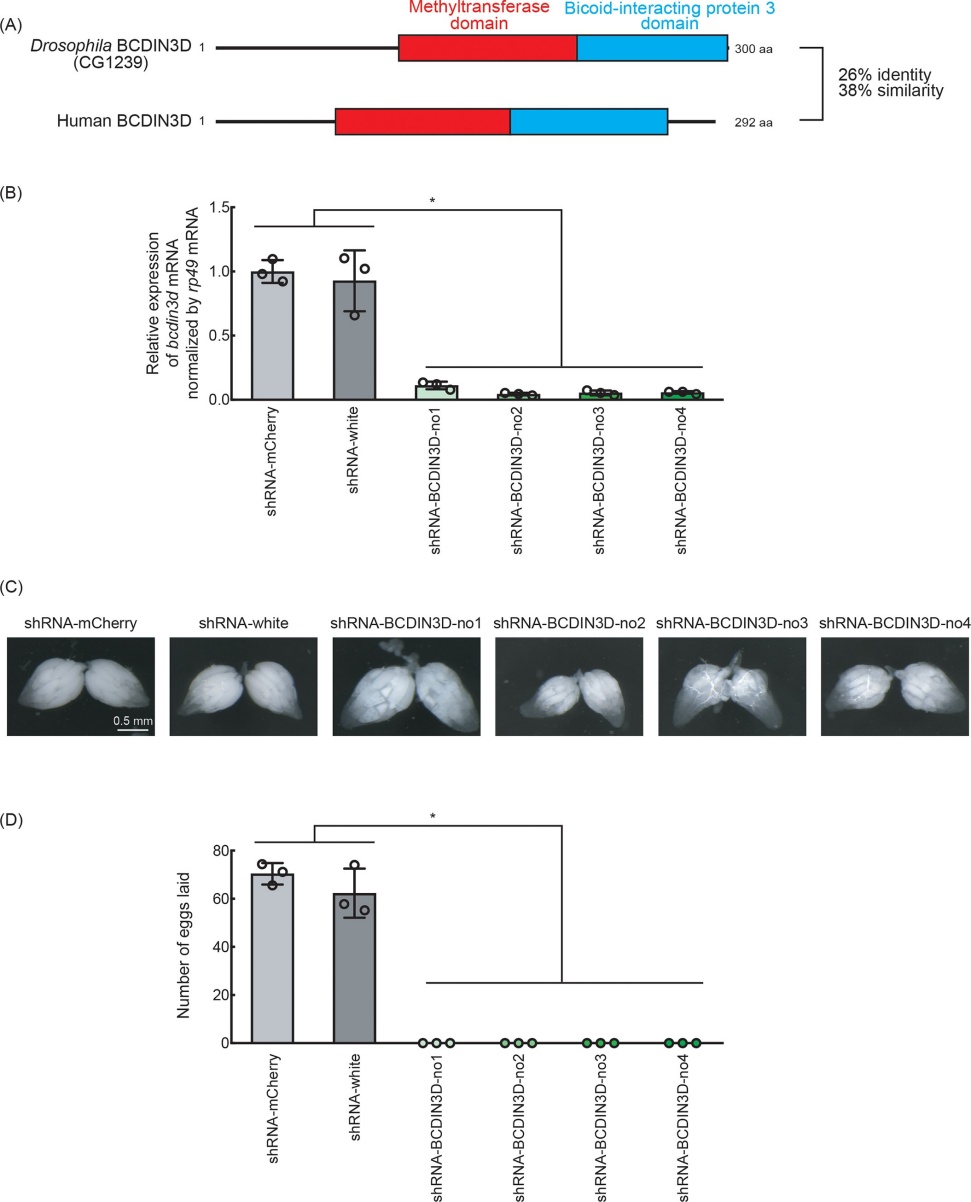
Ovary-specific BCDIN3D knockdown flies cannot lay eggs. (A) Domain structures of *Drosophila* BCDIN3D and human BCDIN3D. (B) Relative abundance of *BCDIN3D* mRNA normalized with *rp49* mRNA in the ovaries of ovary-specific BCDIN3D knockdown and control knockdown flies. Mean ± SD for three biological replicates. P-value <0.05 is indicated by * (Student's t-test). (C) Stereomicroscope images of ovaries dissected from control and BCDIN3D knockdown flies. Scale bar shows 0.5 mm. (D) The numbers of eggs laid by control and BCDIN3D knockdown females crossed with OregonR wild-type males. Mean ± SD for three biological replicates. Each biological replicate datum represents the average number of eggs laid per single female in the cage containing 5 females and 3 males. P-value <0.05 is indicated by * (Student's t-test).

Human RNA methyltransferase BCDIN3D (Bicoid interacting 3 domain containing RNA methyltransferase) contains a methyltransferase domain and a Bicoid-interacting protein 3 domain (Fig 1A). It was previously reported that BCDIN3D specifically di-methylates 5′-monophosphate of pre-miR-145 using SAM as a methyl donor [9]. Dimethylation of 5′-monophosphate of pre-miR-145 blocks its cleavage by the miRNA generating enzyme Dicer, preventing production of miR-145. It was also reported that BCDIN3D specifically mono-methylates the 5′-monophosphate of cytoplasmic tRNA^His^ [10]. Functional consequence of the monomethylation of tRNA^His^ remains unknown. In addition, BCDIN3D mRNA is overexpressed in human breast cancer cells [11] and elevated expression of BCDIN3D mRNA has been associated with poor prognosis in breast cancer [12]. BCDIN3D depletion in breast cancer cells abolishes their tumorigenic phenotypes [9]. Although BCDIN3D is conserved from worm to human, the biological functions of human BCDIN3D and its orthologs remain unknown. Moreover, molecular functions of BCDIN3D orthologs also remain elusive.

CG1239 is a *Drosophila* ortholog of BCDIN3D, and thus we name it *Drosophila* BCDIN3D (Fig 1A). *Drosophila* BCDIN3D and human BCDIN3D share 26% identity and 38% similarity in their amino acid sequences. Here, we studied *Drosophila* BCDIN3D to gain insights into its biological and molecular functions. Ovary-specific depletion of BCDIN3D caused complete female sterility. High-throughput sequencing showed dysregulation of miRNA and mRNA profiles in BCDIN3D knockdown ovaries. Pathway analysis suggested dysregulated pathways such as those involved in metabolic processes, ribonucleoprotein complex regulation, and translational control. Our results reveal that *Drosophila* BCDIN3D plays crucial roles in female fertility and contributes to normal miRNA and mRNA profiles in ovaries.

## Results

### Ovary-specific knockdown of BCDIN3D causes female sterility

To investigate the effects of loss of BCDIN3D function in vivo, we generated BCDIN3D knockout alleles using the CRISPR-cas9 system [13]. Both homozygous and trans-heterozygous BCDIN3D knockouts resulted in embryonic lethality. Since we could not generate complete BCDIN3D null flies because of the embryonic lethality, we chose to knockdown BCDIN3D expression using RNAi. As BCDIN3D is most highly expressed in the ovary (FlyBase. http://flybase.org/reports/FBgn0037368), we knocked down BCDIN3D expression specifically in ovaries using short hairpin RNAs driven by the ovary-specific mata-GAL4-VP16 driver. We tested four different BCDIN3D knockdown strains: shRNA-BCDIN3D-no1, shRNA-BCDIN3D-no2, shRNA-BCDIN3D-no3, and shRNA-BCDIN3D-no4. As controls, we used shRNA-mCherry and shRNA-white. Using qRT-PCR, we confirmed efficient knockdown of *BCDIN3D* expression (89–96% depletion) in ovaries of shRNA-BCDIN3D-no1, shRNA-BCDIN3D-no2, shRNA-BCDIN3D-no3, and shRNA-BCDIN3D-no4 flies, relative to the control shRNA-mCherry and shRNA-white flies (Fig 1B).

BCDIN3D knockdown ovaries did not have dramatically different morphologies compared with control knockdown ovaries (Fig 1C). However, none of the BCDIN3D knockdown flies could lay eggs while control knockdown flies laid ~60–70 eggs per day per female (Fig 1D). These results revealed that BCDIN3D is required for female fertility.

### Ovary-specific knockdown of BCDIN3D causes dysregulation of miRNA profile

**Fig 2:**
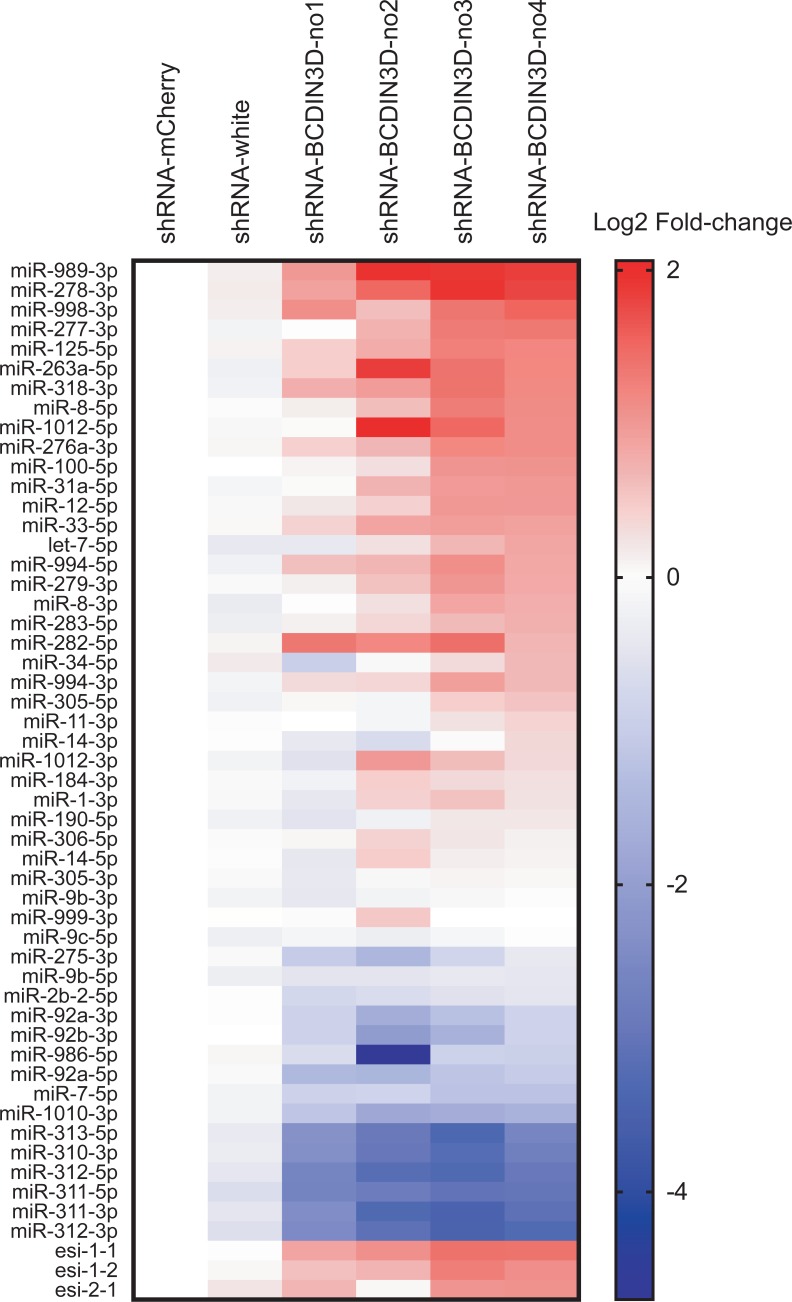
Ovary-specific knockdown of BCDIN3D causes dysregulation of miRNA profile in ovaries. Heatmap of normalized miRNA and endo-siRNA (esi-1.1, esi-1.2, and esi-2.1) abundance in control and BCDIN3D knockdown ovaries determined by high-throughput sequencing. Mean log2 fold-change of three biological replicates relative to shRNA-mCherry control is shown. miRNAs are sorted in descending order of log2 fold-change in shRNA-BCDIN3D-no4. To eliminate miRNAs with very low expression levels, only miRNAs (n = 50) whose normalized mean abundance was more than 100 reads per million total reads in either of the strains are shown.

To gain insight into the molecular mechanism by which ovary-specific knockdown of BCDIN3D causes female sterility, we performed high-throughput sequencing of ovary small RNAs (sRNA-seq) using three biological replicates for each genotype (S1 Table). We found that BCDIN3D knockdown causes dysregulation of miRNA profile in ovaries. Some miRNAs are significantly upregulated and others are downregulated in BCDIN3D knockdown ovaries compared with control knockdown ovaries (Fig 2). Endo-siRNAs (esi-1.1, esi-1.2, and esi-2.1) were generally upregulated in BCDIN3D knockdown ovaries. The trends of the miRNA and siRNA dysregulation were similar among the four different BCDIN3D shRNA knockdown flies, showing reproducibility.

**Fig 3:**
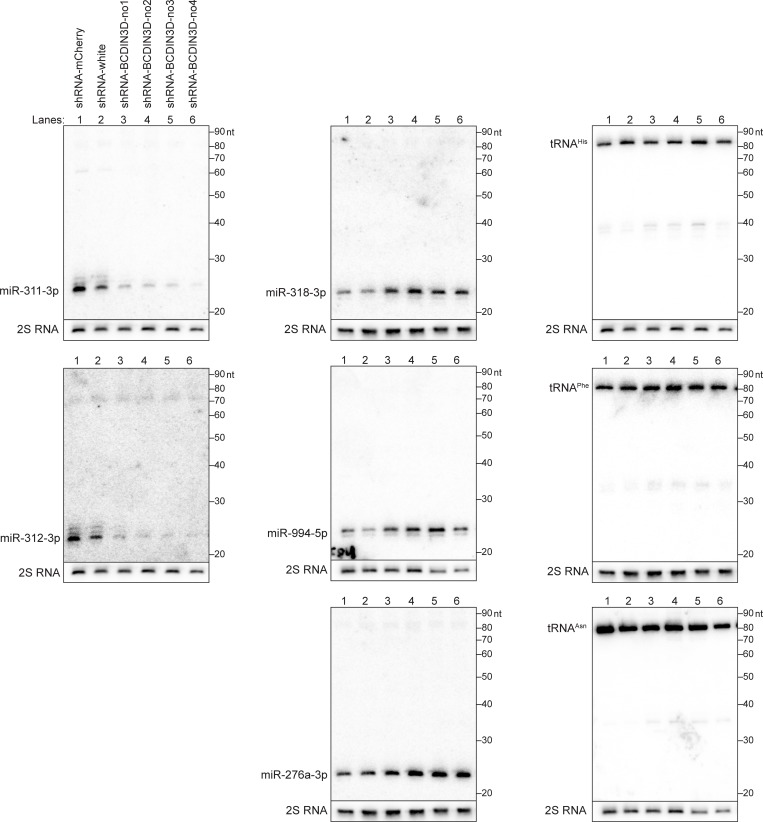
Northern blots of miRNAs and tRNAs confirm dysregulation of miRNA profiles. Representative images of Northern blots of selected miRNAs and tRNAs in control and BCDIN3D knockdown ovaries. Quantification is shown in Fig 4.

**Fig 4:**
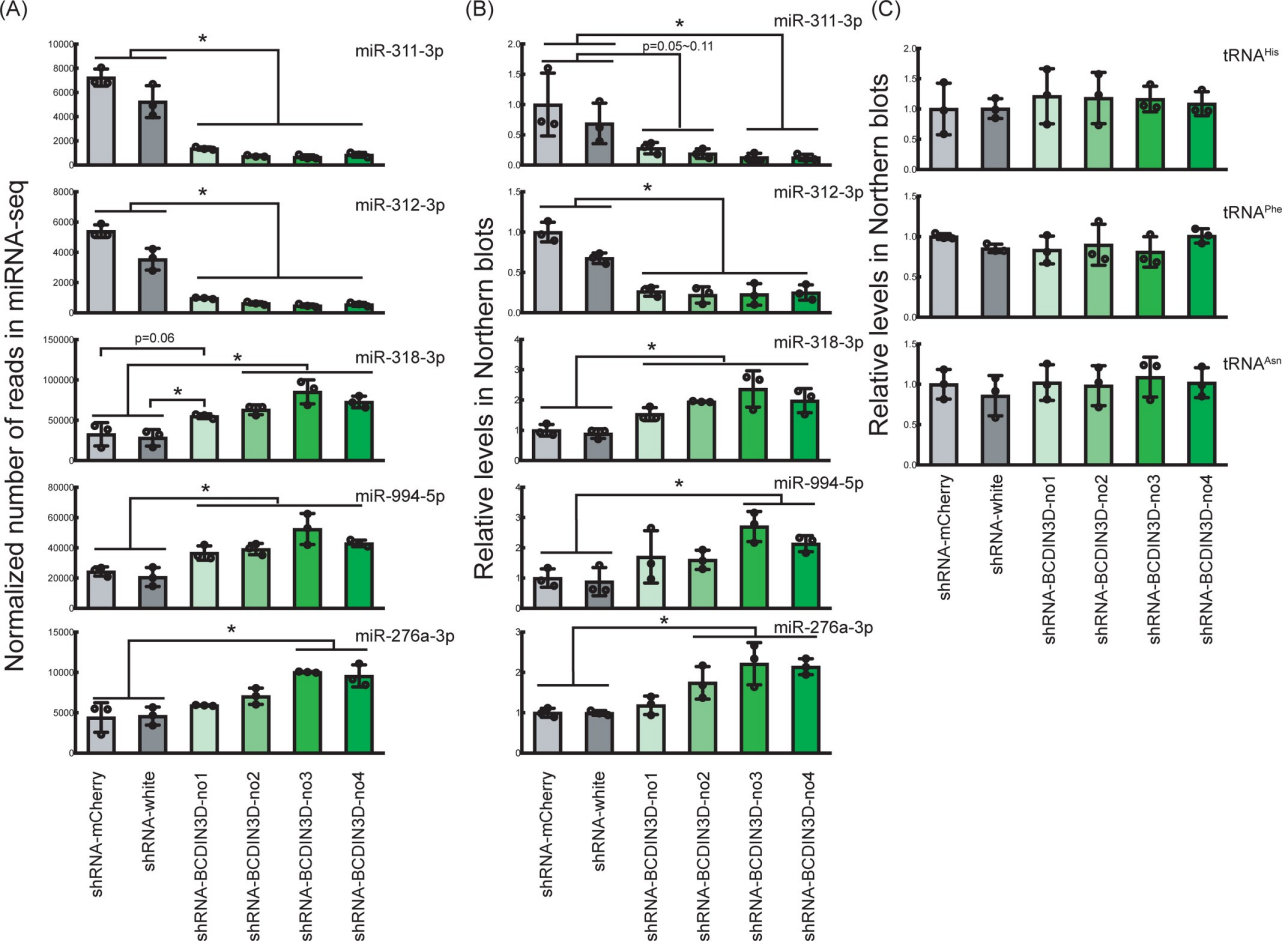
Abundance of dysregulated miRNAs determined by sRNA-seq and Northern blots. (A) Normalized number of reads of miRNAs in control and BCDIN3D knockdown ovaries determined by sRNA-seq. (B) Relative abundance of miRNAs in control and BCDIN3D knockdown ovaries determined by Northern blots. Representative images are shown in Fig 3. (C) Relative abundance of tRNAs in control and BCDIN3D knockdown ovaries determined by Northern blots. Representative images are shown in Fig 3. Mean ± SD for three biological replicates. P-value <0.05 is indicated by * (Student's t-test).

To confirm dysregulation of miRNA profiles in BCDIN3D knockdown ovaries, we performed Northern blots to examine levels of individual miRNAs. Consistent with the sRNA-seq results (Fig 2), miR-311-3p and miR-312-3p were downregulated in BCDIN3D knockdown ovaries compared with control ovaries (Figs 3 and 4). In contrast, miR-318-3p, miR-994-5p, and miR-276a-3p were upregulated in BCDIN3D knockdown ovaries compared with control ovaries (Figs 3 and 4). We did not observe bands corresponding to precursors of these miRNAs (pre-miRNAs), which are ~60–80 nt long (Fig 3). These results show that the abundance of these miRNAs is changed by BCDIN3D knockdown without a clear change in that of corresponding pre-miRNAs.

As human BCDIN3D was reported to mono-methylates the 5′-monophosphate of tRNA^His^ [10], we also examined abundance of a few tRNAs (tRNA^His^, tRNA^Phe^, and tRNA^Asn^) by Northern blots. We observed no significant change in levels of tRNA^His^, tRNA^Phe^, and tRNA^Asn^ in BCDIN3D knockdown ovaries compared with control ovaries (Figs 3 and 4).

### Ovary-specific knockdown of BCDIN3D causes dysregulation of mRNA profile

**Fig 5:**
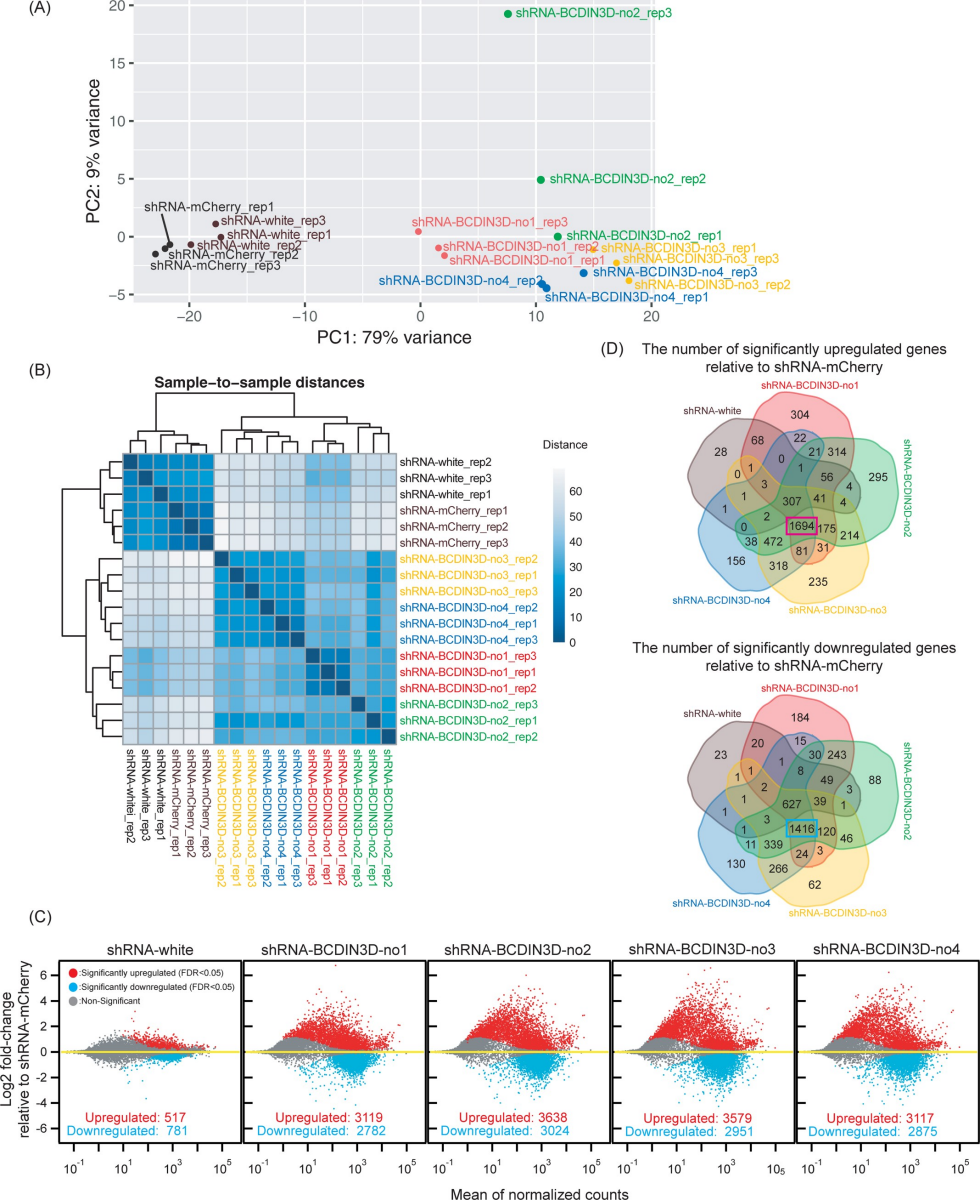
Ovary-specific knockdown of BCDIN3D causes dysregulation of mRNA profile in ovaries. (A) Principal component analysis of mRNA profiles in ovaries determined by mRNA-seq. Three biological replicates each [rep1, rep2, rep3] for each genotype were tested.(B) Sample-to-sample distance matrix of mRNA profiles in ovaries determined by mRNA-seq.(C) MA plots of mRNA expression. x-axis shows mean of normalized counts among the 6 biological samples tested (3 biological replicates each for two genotypess compared in each graph). y-axis shows log2 fold-change in the normalized counts relative to shRNA-mCherry control. (D) Venn diagrams of the numbers of significantly (FDR<0.05) upregulated genes (top) and downregulated genes (bottom) relative to shRNA-mCherry control.

Next, we performed high-throughput sequencing of poly-A+ mRNAs (mRNA-seq) using three biological replicates for each genotype (S2 Table). Principal component analysis and sample-to-sample distance analysis showed that mRNA profiles in BCDIN3D knockdown (four different fly strains [shRNA-BCDIN3D-no1, shRNA-BCDIN3D-no2, shRNA-BCDIN3D-no3, shRNA-BCDIN3D-no4], three biological replicates each [rep1, rep2, rep3]) clustered together, away from those in control knockdown (two different strains [shRNA-mCherry, shRNA-white], three biological replicates each) (Fig 5A and 5B). We examined differential expression of ~17,000 individual genes (Fig 5C). Only 517 genes were significantly (FDR<0.05) upregulated and 781 genes were downregulated in shRNA-white compared with shRNA-mCherry. In contrast, ~3000 genes were upregulated and ~3000 genes were downregulated in each of the four BCDIN3D knockdown strains compared with shRNA-mCherry. Venn diagram analysis showed that among them, as many as 1694 genes were significantly upregulated and 1416 genes were downregulated in all of the four BCDIN3D knockdown strains but not in shRNA-white, relative to shRNA-mCherry, showing reproducibility in the pattern of mRNA dysregulation (Fig 5D).

**Fig 6:**
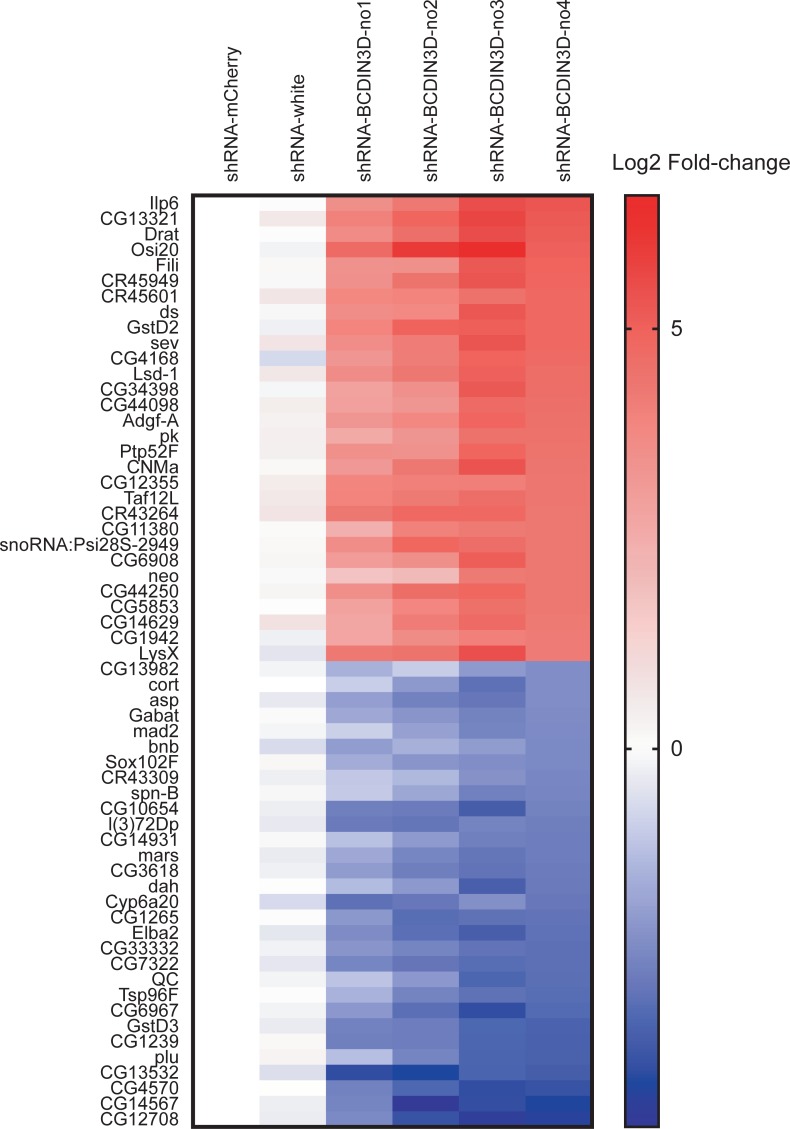
Top30 upregulated and downregulated genes. Heatmap of relative abundance of top 30 upregulated and top 30 downregulated genes determined by mRNA-seq. Mean log2 fold-change of three biological replicates relative to shRNA-mCherry control is shown. Genes are sorted in descending order of log2 fold-change in shRNA-BCDIN3D-no4.

The top 30 upregulated and top 30 downregulated genes (based on the differential expression in shRNA-BCDIN3D-no4) exhibit reproducible patterns of mRNA dysregulation among the four BCDIN3D knockdown strains (Fig 6). The top five upregulated genes were *Ilp6* (Insulin-like peptide 6), *CG13321*, *Drat* (Death resistor Adh domain containing target), *Osi20* (Osiris 20), and *Fili* (Fish-lips). The top five downregulated genes were *CG12708*, *CG14567*, *CG4570*, *CG13532*, and *plu* (plutonium).

### GO term pathway enrichment analysis of differentially expressed genes

1694 genes were significantly upregulated and 1416 genes were downregulated consistently in BCDIN3D knockdown ovaries compared with control knockdown ovaries. Using these sets of differentially expressed genes, we performed GO term pathway enrichment analysis to predict the pathways dysregulated in BCDIN3D knockdown ovaries.

GO term analysis of the 1694 upregulated genes revealed high enrichment for GO term biological processes related with ribonucleoprotein complex (i.e. ribonucleoprotein complex assembly and ribonucleoprotein), translation (i.e. formation of translation initiation ternary complex, translational termination, translational elongation, translation, cytoplasmic translation) and RNAs (i.e. ncRNA metabolic process, rRNA metabolic process, rRNA processing, RNA metabolic process) (Table 1). GO term molecular functions related with ribosomes and RNA binding (i.e. structural constituent of ribosome, RNA binding, rRNA binding) were enriched. GO term cellular components related with ribosome (i.e. cytosolic large ribosomal subunit, ribosomal subunit, large ribosomal subunit, cytosolic small ribosomal subunit, preribosome, ribonucleoprotein complex, mitochondrial large ribosomal subunit) were highly enriched.

**Table 1:** GO term enrichment analysis of upregulated genes.

PANTHER GO-Slim Biological Process	P-value
ribonucleoprotein complex assembly (GO:0022618)	7.72E-11
ribonucleoprotein complex subunit organization (GO:0071826)	1.22E-10
protein-containing complex subunit organization (GO:0043933)	5.78E-10
formation of translation initiation ternary complex (GO:0001677)	6.75E-10
translational termination (GO:0006415)	6.75E-10
translational elongation (GO:0006414)	6.75E-10
cellular protein complex disassembly (GO:0043624)	1.96E-09
protein-containing complex disassembly (GO:0032984)	2.55E-09
ncRNA metabolic process (GO:0034660)	1.27E-07
rRNA metabolic process (GO:0016072)	5.02E-07
rRNA processing (GO:0006364)	2.51E-06
cellular component organization or biogenesis (GO:0071840)	1.62E-05
cellular component organization (GO:0016043)	1.21E-04
organic substance biosynthetic process (GO:1901576)	1.87E-03
biosynthetic process (GO:0009058)	2.70E-03
translation (GO:0006412)	2.84E-03
cellular macromolecule biosynthetic process (GO:0034645)	3.43E-03
macromolecule biosynthetic process (GO:0009059)	5.30E-03
cytoplasmic translation (GO:0002181)	5.55E-03
RNA metabolic process (GO:0016070)	5.68E-03
nucleobase-containing compound metabolic process (GO:0006139)	9.69E-03
cell surface receptor signaling pathway (GO:0007166)	9.79E-03
cellular aromatic compound metabolic process (GO:0006725)	1.06E-02
nucleic acid metabolic process (GO:0090304)	1.48E-02
cellular component biogenesis (GO:0044085)	4.01E-02
PANTHER GO-Slim Molecular Function	P-value
structural constituent of ribosome (GO:0003735)	4.50E-21
structural molecule activity (GO:0005198)	1.93E-13
binding (GO:0005488)	5.40E-05
RNA binding (GO:0003723)	6.98E-05
rRNA binding (GO:0019843)	9.76E-03
PANTHER GO-Slim Cellular Component	P-value
cytoplasmic part (GO:0044444)	1.26E-19
cytosolic large ribosomal subunit (GO:0022625)	9.19E-14
intracellular part (GO:0044424)	1.05E-13
ribosomal subunit (GO:0044391)	2.18E-13
large ribosomal subunit (GO:0015934)	2.18E-13
cell (GO:0005623)	1.18E-12
cell part (GO:0044464)	1.46E-12
cytosolic part (GO:0044445)	5.81E-10
cytosolic small ribosomal subunit (GO:0022627)	1.59E-09
preribosome (GO:0030684)	2.92E-07
small-subunit processome (GO:0032040)	6.73E-05
t-UTP complex (GO:0034455)	7.25E-05
ribonucleoprotein complex (GO:1990904)	1.37E-04
mitochondrial large ribosomal subunit (GO:0005762)	3.10E-03
actin cytoskeleton (GO:0015629)	7.05E-03
mitochondrial protein complex (GO:0098798)	1.02E-02
mitochondrial part (GO:0044429)	1.47E-02
DNA-directed RNA polymerase I complex (GO:0005736)	4.27E-02
preribosome, large subunit precursor (GO:0030687)	4.45E-02

Significantly enriched GO terms associated with the 1694 genes identified in Fig 5D, that are significantly upregulated in shRNA-BCDIN3D-no1, shRNA-BCDIN3D-no2, shRNA-BCDIN3D-no3, and shRNA-BCDIN3D-no4, but not in shRNA-white, relative to shRNA-mCherry.

GO term analysis of the 1416 downregulated genes revealed high enrichment for GO term biological processes related with metabolism (i.e. cellular macromolecule metabolic process, DNA metabolic process, macromolecule metabolic process, metabolic process, organic substance metabolic process, and regulation of metabolic process) and cell cycles (i.e. nuclear division, regulation of cell cycle, cell cycle process, mitotic nuclear division, cell cycle, meiotic nuclear division, cell cycle phase transition, spindle organization, chromosome segregation) (Table 2). GO term molecular functions related with binding and catalytic activity were enriched. Various GO term cellular components were enriched. Dysregulation of these pathways may cause the female sterility by ovary-specific BCDIN3D knockdown.

**Table 2:** GO term enrichment analysis of downregulated genes.

PANTHER GO-Slim Biological Process
cellular macromolecule metabolic process (GO:0044260)
DNA metabolic process (GO:0006259)
macromolecule metabolic process (GO:0043170)
metabolic process (GO:0008152)
organic substance metabolic process (GO:0071704)
organelle organization (GO:0006996)
nuclear division (GO:0000280)
organelle fission (GO:0048285)
cellular process (GO:0009987)
regulation of metabolic process (GO:0019222)
regulation of biological process (GO:0050789)
regulation of cell cycle (GO:0051726)
biological regulation (GO:0065007)
cell cycle process (GO:0022402)
DNA biosynthetic process (GO:0071897)
cellular component organization (GO:0016043)
regulation of macromolecule metabolic process (GO:0060255)
biosynthetic process (GO:0009058)
cellular component organization or biogenesis (GO:0071840)
macromolecule biosynthetic process (GO:0009059)
mitotic nuclear division (GO:0140014)
organic substance biosynthetic process (GO:1901576)
cellular macromolecule biosynthetic process (GO:0034645)
DNA repair (GO:0006281)
cell cycle (GO:0007049)
transcription, DNA-templated (GO:0006351)
regulation of gene expression (GO:0010468)
DNA replication (GO:0006260)
regulation of cellular macromolecule biosynthetic process (GO:2000112)
regulation of macromolecule biosynthetic process (GO:0010556)
regulation of biosynthetic process (GO:0009889)
macromolecule modification (GO:0043412)
meiotic nuclear division (GO:0140013)
regulation of organelle organization (GO:0033043)
cell cycle phase transition (GO:0044770)
regulation of mitotic cell cycle (GO:0007346)
transcription by RNA polymerase II (GO:0006366)
regulation of mitotic cell cycle phase transition (GO:1901990)
DNA replication initiation (GO:0006270)
spindle organization (GO:0007051)
chromosome segregation (GO:0007059)
chromosome organization (GO:0051276)
PANTHER GO-Slim Molecular Function
binding (GO:0005488)
catalytic activity (GO:0003824)
catalytic activity, acting on a protein (GO:0140096)
protein binding (GO:0005515)
protein kinase activity (GO:0004672)
DNA binding (GO:0003677)
pyrophosphatase activity (GO:0016462)
hydrolase activity, acting on acid anhydrides, in phosphorus-containing anhydrides (GO:0016818)
hydrolase activity, acting on acid anhydrides (GO:0016817)
organic cyclic compound binding (GO:0097159)
nucleic acid binding (GO:0003676)
helicase activity (GO:0004386)
nucleoside-triphosphatase activity (GO:0017111)
enzyme binding (GO:0019899)
microtubule binding (GO:0008017)
DNA helicase activity (GO:0003678)
protein serine/threonine kinase activity (GO:0004674)
chromatin binding (GO:0003682)
tubulin binding (GO:0015631)
cytoskeletal protein binding (GO:0008092)
DNA replication origin binding (GO:0003688)
hydrolase activity (GO:0016787)
peptide N-acetyltransferase activity (GO:0034212)
cyclin-dependent protein kinase activity (GO:0097472)
cyclin-dependent protein serine/threonine kinase activity (GO:0004693)
double-stranded DNA binding (GO:0003690)
peptide-lysine-N-acetyltransferase activity (GO:0061733)
cysteine-type endopeptidase activity (GO:0004197)
acetyltransferase activity (GO:0016407)
histone binding (GO:0042393)
PANTHER GO-Slim Cellular Component
intracellular part (GO:0044424)
protein-containing complex (GO:0032991)
cell part (GO:0044464)
cell (GO:0005623)
catalytic complex (GO:1902494)
transferase complex (GO:1990234)
intracellular organelle part (GO:0044446)
nucleus (GO:0005634)
intracellular membrane-bounded organelle (GO:0043231)
membrane-bounded organelle (GO:0043227)
organelle (GO:0043226)
transferase complex, transferring phosphorus-containing groups (GO:0061695)
cytoplasm (GO:0005737)
nuclear part (GO:0044428)
chromosomal part (GO:0044427)
nucleoplasm part (GO:0044451)
CMG complex (GO:0071162)
serine/threonine protein kinase complex (GO:1902554)
protein kinase complex (GO:1902911)
MCM core complex (GO:0097373)
MCM complex (GO:0042555)
GINS complex (GO:0000811)
DNA replication preinitiation complex (GO:0031261)
protein-DNA complex (GO:0032993)
origin recognition complex (GO:0000808)
nuclear pre-replicative complex (GO:0005656)
pre-replicative complex (GO:0036387)
nuclear chromosome part (GO:0044454)

Significantly enriched GO terms associated with the 1416 genes identified in Fig 5D, that are significantly downregulated in shRNA-BCDIN3D-no1, shRNA-BCDIN3D-no2, shRNA-BCDIN3D-no3, and shRNA-BCDIN3D-no4, but not in shRNA-white, relative to shRNA-mCherry.

## Discussion

In this study, we show that ovary-specific knockdown of *Drosophila* BCDIN3D causes female sterility without clear morphological defect in ovaries (Fig 1). High-throughput sequencing studies demonstrated that BCDIN3D plays important roles in defining miRNA and mRNA profiles in ovaries (Figs 2–6).

Some miRNAs were upregulated and other miRNAs were downregulated in BCDIN3D knockdown ovaries (Figs 2–4). Previous studies showed that human BCDIN3D reduces miR-145 level by di-methylating the 5′-monophosphate of pre-miR-145, which prevents its processing by Dicer [9]. If *Drosophila* BCDIN3D similarly di-methylates certain pre-miRNAs and thereby blocks the production of the corresponding miRNAs, BCDIN3D knockdown is expected to cause upregulation of such miRNAs. We found that BCDIN3D knockdown causes upregulation of miR-318-3p, miR-994-5p, and miR-276a-3p (Figs 3 and 4). In contrast, we did not observe accumulation of pre-miR-318, pre-miR-994, and pre-miR-276a in control knockdown ovaries compared with BCDIN3D knockdown ovaries in our Northern blots (Fig 3). Thus, if these pre-miRNAs (pre-miR-318, pre-miR-994, and pre-miR-276a) are BCDIN3D substrates and are di-methylated blocking Dicer cleavage, such di-methylated pre-miRNAs might be degraded by other RNases rather than stably accumulated.

Other previous study showed that human BCDIN3D mono-methylates the 5′-monophosphate of tRNA^His^ [10]. The presence or absence of monomethylation did not affect the steady state level of tRNA^His^ in culture cells and therefore the molecular and biological functions of this tRNA^His^ monomethylation remain unknown. We found that the levels of tRNA^His^, tRNA^Phe^, and tRNA^Asn^ are not changed in BCDIN3D knockdown ovaries. This implies that, if BCDIN3D mono-methylates tRNA^His^, the modification does not affect steady-state stability of tRNA^His^ in *Drosophila* ovaries either. Further study should examine whether *Drosophila* BCDIN3D methylates candidate pre-miRNAs and tRNA^His^.

We identified tens of miRNAs and thousands of mRNAs whose expression level is dysregulated upon BCDIN3D knockdown. Unbiased, transcriptome-wide approach to determine RNAs that are directly bound and methylated by *Drosophila* BCDIN3D and characterizing RNA methylation profiles between control and BCDIN3D knockdown ovaries will provide useful insight on the mechanisms by which BCDIN3D regulates these RNA levels.

In this study, we showed for the first time the biological function of evolutionary conserved RNA methyltransferase BCDIN3D in *Drosophila* female fertility. We also showed that *Drosophila* BCDIN3D is important for normal miRNA and mRNA profiles in ovaries. Our results indicate that RNA methylation by BCDIN3D plays crucial biological and molecular functions.

## Materials and methods

### Fly strains

shRNA-BCDIN3D-no1 plasmid construct was created by inserting dsDNA fragment comprised of oligo1 (5’-CTAGCAGTCGGCAACTATAAGCACTACTATAGTTATATTCAAGCATATAGTAGTGCTTATAGTTGCCGGCG-3’) and oligo2 (5’-AATTCGCCGGCAACTATAAGCACTACTATATGCTTGAATATAACTATAGTAGTGCTTATAGTTGCCGACTG-3’) into the pValium20 plasmid vector linearized with NheI and EcoRI [14] and was integrated at the attP40 site on the position 25C6 on the second chromosome of fly using the PhiC31 system [15]. shRNA-mCherry, shRNA-white, shRNA-BCDIN3D-no2, shRNA-BCDIN3D-no3, and shRNA-BCDIN3D-no4 strains are all pValium20-based lines from Bloomington Drosophila Stock Center (BDSC) with BDSC numbers 35785, 33623, 44506, 42912, and 44023, respectively. Ovary-specific expression of the hairpin RNAs was induced by crossing these strains with the matα4-GAL4-VP16 driver strain. Dissected ovary images were taken using Leica M125 Stereomicroscope.

### Fertility assay

For the female fertility assay, five test virgin females were mated with three wild-type (OregonR) males in a cage with a 6-cm grape juice agar plate with wet yeast paste (Genesee) [16–18]. The grape juice agar plate was exchanged with a fresh one every day. The number of eggs laid on the third grape juice agar plate (set up on day 3 and recovered on day 4) was counted.

### RNA preparation

Ovaries were hand-dissected from 2–5 days old flies fed with wet yeast paste [19]. Total RNA from ovaries was purified using miRVana (ThermoFisher). Tens of ovaries were used for each biological replicate preparation of the ovary RNA, resulting in tens to hundreds of microgram of purified total ovary RNAs.

### qRT-PCR

qRT-PCR was performed as previously described [17, 20–22]. RNAs were treated with Turbo DNase (Thermo Fisher Scientific) and were reverse-transcribed into cDNA using an oligo-dT primer and AMV Reverse Transcriptase (NEB). qPCR was performed using SsoAdvanced Universal SYBR Green Supermix on CFX96 (Biorad) and results were analyzed using CFX Manager (Biorad). The primers used were as follows. *BCDIN3D*, 5’-ACCGCAAGATGTGGTAAACA-3’ and 5’-TCCATTGTGAAATCCAAAGTTACA-3’. *rp49*, 5’-CTGCCCACCGGATTCAAG-3’ and 5’-CGATCTCGCCGCAGTAAAC-3’.

### Northern blot

Northern blot was performed as described previously [16, 23]: 12 μg total ovary RNA was denatured in formamide loading buffer (98% v/v formamide, 0.1% w/v bromophenol blue and xylene cyanol, 10mM EDTA) at 95 C for 5 min and was resolved on a 0.4 mm thick, 12% denaturing polyacrylamide 7 M urea sequencing gel in 0.5×TBE (Tris-Borate-EDTA) buffer. After electrophoresis, RNA was transferred at 20 V for 1 hr to a Hybond-N+ membrane (GE healthcare) in 0.5×TBE buffer using a semi-dry transfer system (Transblot SD, Bio-Rad). The RNA was UV cross-linked (HL2000, UVP) to the membrane and pre-hybridized in Church buffer for at least 60 min at 37 C. DNA oligonucleotide probes were 5′ ^32^P-radiolabeled with T4 polynucleotide kinase (NEB) and γ-^32^P-ATP. After labelling, non-incorporated nucleotides were removed using a Sephadex G-25 spin column (GE healthcare) and the probes were added to the Church buffer and hybridized for at least 6 hrs at 37 C. Membranes were washed three times for 10 min in 2×SSC containing 0.05% (w/v) SDS at 37 C, subsequently exposed to Storage Phosphor Screens (GE healthcare) and analyzed using FLA-9500 (GE healthcare). Probes were stripped from the membranes in boiling 0.1% SDS solution. The membranes were re-probed with the next probe. The DNA oligo probes used were as follows.

miR-275-3p, 5’-CGCGCGCTACTTCAGGTACCTGA-3’.

miR-276a-3p, 5’-AGAGCACGGTATGAAGTTCCTA-3’.

miR-279-3p, 5’-TTAATGAGTGTGGATCTAGTCA-3’.

miR-311-3p, 5’-TCAGGCCGGTGAATGTGCAATA-3’.

miR-312-3p, 5’-TCAGGCCGTCTCAAGTGCAATA-3’.

miR-318-3p, 5’-TGAGATAAACAAAGCCCAGTGA-3’.

miR-994-5p, 5’-ATCACGGCTACTATTTCCTTA-3’.

tRNA^His^, 5’-CGTGGGGTCCTAACCACTAGACGATCACGGC-3’.

tRNA^Phe^, 5’-TCTAACGCTCTCCCAACTGAGCTATTTCGGC-3’

tRNA^Asn^, 5’-CGAACGCGCTAACCAATTGCGCCACGGAGGC-3’

2S RNA, 5’-TACAACCCTCAACCATATGTAGTCCAAGC-3’.

### High-throughput sequencing of small RNAs (small RNA-seq)

Three biological replicates each of small RNA libraries were prepared using size-selected 18–30 nt long RNAs by gel purification, sequenced using HiSeq2500 platform (Illumina), and analyzed as previously described [16, 17, 19–22, 24–28].

Approximately 10–19 million reads were obtained for each library. Approximately 62–89% of the reads were mapped to the dm6 *Drosophila* genome. Approximately 11–30% of the non-rRNA-mapping reads were mapped to miRNA hairpins. The abundance of miRNAs normalized by the sequencing depth (non-rRNA-mapping reads) in each library was calculated. Then their mean abundance among the three biological replicates was calculated. Further normalization was performed using quantitative results of the Northern blots for miR-275-3p, miR-276a-3p, miR-279-3p, miR-311-3p, miR-312-3p, miR-318-3p, miR-994-5p, normalized by 2S RNA. To eliminate miRNAs with very low expression levels, which are unlikely to have a physiological role, only miRNAs (n = 50) whose mean abundance was more than 100 reads per million total reads in either of the genotypes and three endo-siRNAs (esi-1.1, esi-1.2, and esi-2.1) were described.

### High-throughput sequencing of mRNAs (mRNA-seq)

Poly-A+ mRNA-seq libraries were constructed as previously described [16, 28, 29]. Paired-end 100 nt sequencing (2x 100 bp) was performed using HiSeq2500 platform (Illumina). Approximately 6–15 million reads were obtained for each library. Approximately 96–98% of the paired reads were mapped to the dm3 *Drosophila* genome using HISAT2 [30] on the Galaxy platform [31]. The differential expression was analyzed using featureCounts [32] and DESeq2 [33] on the Galaxy platform [34, 35]. GO term and KEGG pathway enrichment analyses were performed with GO Enrichment Analysis (http://geneontology.org/page/go-enrichment-analysis) [36] using significantly differentially expressed genes.

### Deposited sequenced libraries

The SRA accession number for the small RNA-seq and mRNA-seq libraries reported in this manuscript is PRJNA518149.

### Statistical test

Unpaired two-tailed Student’s t-test was performed to determine statistical significance. The Benjamini-Hochberg method was used to adjust P-values for multiple comparisons.

## Supporting information

S1 Table Normalized number of reads of miRNAs and endo-siRNAs in sRNA-seq. (XLSX)

S2 Table Differential expression analysis of mRNA-seq. (XLSX)
